# Dietary riboflavin intake in relation to psychological disorders in Iranian adults: an observational study

**DOI:** 10.1038/s41598-023-32309-w

**Published:** 2023-03-29

**Authors:** Parisa Rouhani, Mohammad Amoushahi, Ammar Hassanzadeh Keshteli, Parvane Saneei, Hamid Afshar, Ahmad Esmaillzadeh, Peyman Adibi

**Affiliations:** 1 Students’ Research Committee, Isfahan University of Medical Sciences, Isfahan, Iran; 2 Department of Community Nutrition, School of Nutrition and Food Science, Nutrition and Food Security Research Center, Isfahan University of Medical Sciences, PO Box 81745-151, Isfahan, Iran; 3 Department of Nutrition, Varastegan Institute for Medical Science, Mashhad University of Medical Sciences, Mashhad, Iran; 4 Nutritional Health Team (NHT), Universal Scientific Education and Research Network (USERN), Isfahan, Iran; 5 Department of Medicine, University of Alberta, Edmonton, AB Canada; 6 Isfahan Gastroenterology and Hepatology Research Center, Isfahan University of Medical Sciences, Isfahan, Iran; 7 Psychosomatic Research Center, Department of Psychiatry, Isfahan University of Medical Sciences, Isfahan, Iran; 8 Obesity and Eating Habits Research Center, Endocrinology and Metabolism Molecular-Cellular Sciences Institute, Tehran, Iran; 9 Endocrinology and Metabolism Research Center, Endocrinology and Metabolism Clinical Sciences Institute, Tehran University of Medical Sciences, Tehran, Iran; 10 Department of Community Nutrition, School of Nutritional Sciences and Dietetics, Tehran University of Medical Sciences, Tehran, Iran

**Keywords:** Psychology, Diseases, Health care, Nutrition

## Abstract

Findings of earlier investigations on association between dietary riboflavin intake and psychological disorders are contradictory. Therefore, the relation between dietary riboflavin intake and depression, anxiety, and psychological distress was assessed in Iranian adults. In this cross-sectional study, dietary intakes of 3362 middle-aged adults were collected using a validated dish-based food frequency questionnaire. Daily intake of riboflavin for each participant was calculated by summing up the amount of riboflavin contents of all foods and dishes. Hospital Anxiety and Depression Scale (HADS) and General Health Questionnaire (GHQ), as validated questionnaires among Iranians, have been applied to assess depression, anxiety, and psychological distress. After considering potential confounders, adults in the top energy-adjusted quartile of riboflavin intake, compared to the bottom quartile, had decreased odds of depression (OR = 0.66; 95%CI 0.49, 0.88), anxiety (OR = 0.64; 95%CI 0.44, 0.94) and high psychological distress (OR = 0.65; 95%CI 0.48, 0.89). Stratified analysis by sex revealed that men in the forth quartile of riboflavin intake, compared with those in the first quartile, had respectively 51 and 55% lower odds of depression (OR = 0.49; 95%CI 0.29, 0.83) and anxiety (OR = 0.45; 95%CI 0.21, 0.95). In women, riboflavin intake was significantly associated with lower odds of psychological distress (OR = 0.67; 95%CI 0.46, 0.98). An inverse relation was observed between dietary riboflavin intake and chance of psychological disorders in Iranian adults. High intake of riboflavin decreased the chance of depression and anxiety in men and high psychological distress in women. More prospective studies are needed to confirm these findings.

## Introduction

The prevalence of mental diseases such as depression, anxiety, and psychological distress has been growing worldwide^1^. These disorders are associated with decreased quality of life and fatality^2,3^. Depression and anxiety have respectively a global prevalence of 4.7% and 7.3%^4,5^. A national survey in Iran showed that 20.0% and 20.8% of adults suffered from anxiety and depression, respectively^6,7^. Depression manifests itself physically and emotionally in a variety of ways, including: feelings of worthlessness, overwhelm, misery, loss of confidence, muscle discomfort, and headaches. All of these symptoms contribute to decreased efficiency and functional capacities of the affected individuals^8^. Anxiety, as another common mental disorder, causes excessive worry, fear and has negative effects on health status, and family relationships^9,10^. Mental disorders exacerbate the consequences of a wide variety of medical conditions, contribute to disability, and increase mortality^11^. Based on previous reports, mental disorders had a high social and economic costs on societies and healthcare systems every year^12,13^. Depression and anxiety contribute between 30 and 50% of the worldwide costs of mental health^14^.

Several non-modifiable and modifiable factors including genetics, environmental factors (such as life events, low social support, financial problems), and lifestyle behaviors, including diet, might play critical roles in incidence of depression and anxiety^15^. Notably, the role of food intakes and dietary patterns in development of mental diseases has been recognized^16–18^. Earlier studies demonstrated inverse associations between consumption of dairy products, vegetables, fruits, olive oil and phytochemicals and depression risk^19,20^. Moreover, inadequate intake of micronutrients including magnesium, potassium, zinc, and B vitamins was positively associated with depression^21^. The association of these micronutrients with mental health might be explained by their contribution to neural function and synthesis of several neurotransmitters^22,23^. Riboflavin, known as vitamin B2, is a member of B-group vitamins. This nutrient, as a water-soluble and heat-stable vitamin and an antioxidant, can be involved in metabolizing macronutrients into glucose^24^. Riboflavin could possibly affect mental state of individuals through its two important coenzymes, flavin adenine dinucleotide (FAD) and flavin mononucleotide (FMN)^25–27^.

Several previous studies have examined the relation between vitamin B6, B12, and folate with depression or anxiety in Iranian adults^28,29^. In case of riboflavin, the association between this vitamin and depression has been investigated in East Asia populations^30–32^; however, to the best of our knowledge, no previous study has investigated the relation between riboflavin consumption and common psychological disorders, especially anxiety and psychological distress, in Iranian adults. Therefore, the present study aimed to examine the association between riboflavin consumption and prevalence of depression, anxiety and psychological distress in a large group of Iranian adults.

## Materials and methods

### Study design and participants

The current cross-sectional study was conducted in a retrospective manner on the Study on the Epidemiology of Psychological-Alimentary Health and Nutrition (SEPAHAN) data^33^. This two-phase study was conducted on a group of general Iranian adults working in 50 health care centers affiliated with Isfahan University of Medical Sciences (IUMS). As shown in Supplementary Fig. 1, in the first phase of SEPAHAN, a comprehensive self-administered questionnaire on socio-demographic characteristics, dietary behaviors, and dietary intakes was delivered to 10,087 individuals aged 18–71 years; 8691 of them returned the completed questionnaires (response rate: 86.16%). In the second phase, another set of questionnaires was distributed among the same participants to obtain data on psychological distress and mental health (response rate: 61.85%). After merging data from these two phases, the completed data of 4669 participants were available. There was no significant difference in demographic data between those who returned completed questionnaires and those who did not.

### Exclusion criteria

Some university teaching hospitals and research centers were not included in order to reduce the conflict of interest in research. In addition, adults with a total energy intake outside of the range of 800 and 4200 kcal/day (as under-reporters and over-reporters), those with missing dietary or psychological data, and those who participated in only one phase of the study, participants who suffer from aggressive disease such as cardiovascular, respiratory, neurologic diseases were excluded. However, individuals with mild metabolic disorders such as abdominal obesity, hyperlipidemia, or hypertension were included in the current study. These exclusions resulted in data of 3362 adults for the current analysis.

### Dietary intake assessment

Long-term dietary intakes were evaluated by a validated Willet-format 106-item dish-based semi-quantitative food frequency questionnaire (DS-FFQ) that was specifically designed for Iranian adults^34^. Detailed information about the design, foods included, and validity of this tool has been published elsewhere^34^. The questionnaire included five different dishes and foods categories: (1) dairy products (dairies, butter and cream, 9 items); (2) mixed dishes (canned or cooked, 29 items); (3) grain products and grains (different types of potato, biscuits, bread, and cakes, 10 items); (4) fruits and vegetables and (22 items); and (5) miscellaneous food items and beverages (including fast foods, nuts, beverages, desserts, and sweets, 36 items). The questionnaire was completed by a self-administered method. A one-page written instruction was provided for participants to complete the questionnaire. Participants were asked to report their dietary intakes of mixed dishes and foods in the previous year according to multiple frequency choices differing from ‘never or less than once a month’ to ‘12 or more times per day’. Considering the portion size of each food or dish in the questionnaire and the reported frequency of that item, all foods and dishes were converted to grams per day, using household measures^35^. Then, all gram/day values were input into the Nutritionist IV software to calculate daily energy and nutrient intakes. Dietary riboflavin intake for each individual was calculated by summing up the amount of vitamin B2 in all foods and dishes. The validation study of this DS-FFQ showed that reasonably valid and reliable dietary intakes could be provided by this tool^34^. Additionally, our prior investigations revealed that this DS-FFQ has an acceptable level of validity and reliability for assessing foods and dietary intakes in relation to various diseases^36–38^.

### Assessment of psychological disorders

Anxiety and depression were defined by the validated Iranian version of Hospital Anxiety and Depression Scale (HADS)^39^. HADS is a 14-item scale which includes two subscales: depression and anxiety. Each item comprises a four-point scale; greater scores suggest higher level of depression and anxiety. Score ranges for depression and anxiety are between 0 and 21. In the current study, values of 8 or higher for each subscale were considered as having depression or anxiety, whereas scores of 0–7 were considered as normal status^39^. A validation study on 167 Iranian adults revealed reasonable validity of the translated version of HADS for measurement of mental health^39^.

Furthermore, psychological distress was screened by the validated Iranian version of General Health Questionnaire (GHQ)^40^. This questionnaire contains 12 items with a 4-point rating scale (less than usual, no more than usual, rather more than usual, or much more than usual). Total score for each individual was calculated through a bimodal method (0-0-1-1) and ranged from 0 to 12; higher scores were related to higher degree of psychological distress. In the current study, having a score of 4 or more was defined as high psychological distress^40^. The validity of this questionnaire was reasonable based on a validation study on 748 Iranian adults^41^.

### Assessment of other variables

Data on confounders including age, sex, marital status, education levels, smoking, number of family members, house possession, disease history, current use of anti-psychotic medications (including sertraline, nortriptyline, fluoxetine, amitriptyline or imipramine, citalopram, and fluvoxamine) and dietary supplements (including intake of iron, calcium, and other dietary supplements) was collected through a self-administered questionnaire.

Data on weight (kg) and height (cm) were measured using a self-reported questionnaire. Body mass index (BMI) was calculated as weight in kilograms divided by the square of height in meters. The validity of the self-reported anthropometric measures was investigated in pilot study on 200 individuals. Correlation coefficients between self-reported height, weight, BMI, and waist circumference (WC) and measured values were respectively 0.83, 0.95, 0.70, and 0.60 (P < 0.001 for all)^42^; these robust correlations indicated that the self-reported values of anthropometric indices could provide valid measures in this population. To determine physical activity level of participants, the validated General Practice Physical Activity Questionnaire (GPPAQ) was applied^43^. Then, participants were categorized into two groups: physically active (≥ 1 h/week) and physically inactive (< 1 h/week).

### Statistical analysis

First, energy-adjusted dietary intake of riboflavin was obtained through the residual method^44^. To examine the association of exposure and outcome of the interest, participants were classified into energy-adjusted quartiles of riboflavin intake. Then, data on socio-demographic variables were reported as mean ± SD or percentage. The differences across quartiles of riboflavin were assessed using analysis of variance (ANOVA) or chi-square test. Analysis of covariance (ANCOVA) was applied to examine dietary intakes of participants across quartiles of riboflavin. Crude and multivariable-adjusted models were used to evaluate the association between riboflavin and psychological health status. Confounding variables were determined based on previously published investigations^33,38^. Age (continuous), sex (male/ female), and energy intake (continuous) were adjusted in the first model. More adjustments were done for physical activity (< 1 h/week/ ≥ 1 h/week), house possession (yes/ no), number of family members (≥ 4/ < 4), history of diabetes (yes/no), smoking (current smoker/former smoker/ non-smoker), marital status (single/widow or divorced/married), education (under diploma/ diploma/ above diploma/ bachelors and above), and anti-psychotic medications intake (yes/ no) and dietary supplements (yes/ no) in the second model. In the third model, intakes of iron (continuous), thiamin (continuous), fat (continuous), and n-3 fatty acids (continuous) were additionally adjusted. In the last model, further adjustment for BMI (continuous) was done. All odds ratios were calculated based on the first quartile of riboflavin intake as the reference category. Quartiles of riboflavin intake were considered as an ordinal variable in the logistic regression models to estimate the trend of odds ratios across these categories. Stratified analyses were done to obtain odds ratios for psychological disorders in different categories of sex (men/women) and BMI (< 25/ ≥ 25 kg/m^2^). All statistical analyses were conducted by the use of SPSS software (version 20; SPSS Inc, Chicago IL). P < 0.05 was considered as significant level.

### Ethical approval and consent to participate

All methods were performed in accordance with the STROBE guidelines and regulations. All methods were carried out in accordance with relevant guidelines and regulations. Each participant signed an informed written consent. The Bioethics Committee of Isfahan University of Medical Sciences has ethically approved the SEPAHAN project (no. 189069).

## Results

The study participants consisted of 3362 middle-aged adults (1959 women and 1403 men) with a mean weight of 68.7 kg. General characteristics of the study population across energy-adjusted quartiles of dietary riboflavin intake are provided in Table 1. Individuals in the fourth quartile of riboflavin intake in comparison to the first quartile were more likely to have type 2 diabetes and obesity, higher age, weight, and BMI.

**Table 1:** General characteristics of study participants across energy-adjusted quartiles of riboflavin intake (n = 3362).

	Quartiles of riboflavin intake				P^a^
	Q1 (n = 840) (< 1.6 mg/d)	Q2 (n = 841) (1.6–1.82 mg/d)	Q3 (n = 841) (1.83–2.09 mg/d)	Q4 (n = 840) (> 2.09 mg/d)	
Age (years)	35.55 ± 7.82	35.92 ± 7.74	36.15 ± 7.80	37.52 ± 7.97	< 0.001
Weight (kg)	68.07 ± 12.79	68.03 ± 14.31	67.99 ± 12.23	70.52 ± 13.16	< 0.001
BMI (kg/m^2^)	24.65 ± 3.72	24.58 ± 3.92	24.87 ± 3.82	25.51 ± 3.74	< 0.001
Female (%)	57.4	57.2	60.8	57.7	0.40
Marital status (%)					0.48
Married	80.3	82.3	80.8	83.3	
Single	18.0	16.2	17.7	14.6	
Divorced/widow	1.7	1.5	1.4	2.1	
Education (%)					0.94
Under diploma	12.4	11.1	12.5	12.2	
Diploma	27.6	27.8	25.4	26.9	
Above diploma and under master's	52.7	53.0	55.2	52.9	
Master's and above	7.3	8.1	6.9	7.9	
Family members (%)					0.18
≤ 4	85.8	86.8	89.3	87.3	
> 4	14.2	13.2	10.7	12.7	
House possession (yes) (%)	52.4	60.9	55.9	63.9	< 0.001
Diabetes (%)	2.3	1.3	1.1	2.5	0.07
Anti-depressants medications use^b^ (%)	5.2	6.7	5.2	5.1	0.46
Dietary supplement use^c^ (%)	29.8	29.0	30.9	30.4	0.85
Smokers (%)	13.3	15.2	12.4	14.3	0.36
Physically activity (%)					0.18
< 1 h/week	85.7	87.5	88.6	85.5	
≥ 1 h/week	14.3	12.5	11.4	14.5	
Obese^d^ (%)	42.6	42.7	42.7	51.2	< 0.001

All values are means ± standard deviation (SD), unless indicated.

^a^Obtained from ANOVA for continuous variables and chi-square test for categorical variables.

^b^Anti-depressants medications include nortriptyline, amitriptyline or imipramine, fluoxetine, citalopram, fluvoxamine and sertraline.

^c^Dietary supplements include iron, calcium, vitamins and other dietary supplements.

^d^BMI ≥ 25(kg/m^2^).

Dietary intakes of selected nutrients and food groups of study participants across energy-adjusted quartiles of dietary riboflavin intake are presented in Table 2. Individuals in the top quartile of riboflavin intake had significantly higher intake of protein, carbohydrate, dietary fiber, thiamin, pyridoxine, iron, vitamin C, whole grain, fruit, vegetable, and dairy (low fat and high fat) in comparison to those in the bottom quartile. Whereas, subjects in the last quartile of riboflavin in comparison to those in the reference quartile had lower consumption of energy, fat, vitamin E, red meat, refined grain, omega-3 fatty acids, nuts, soy and legumes.

**Table 2:** Dietary intakes of selected nutrients and food groups across energy-adjusted quartiles of riboflavin intake (n = 3362).

	Quartiles of riboflavin intake				P^a^
	Q1 (n = 840) (< 1.6 mg/d)	Q2 (n = 841) (1.6–1.82 mg/d)	Q3 (n = 841) (1.83–2.09 mg/d)	Q4 (n = 840) (> 2.09 mg/d)	
Energy (kcal/d)	2559.29 ± 29.88	2249.15 ± 29.37	2229.52 ± 29.12	2499.72 ± 29.33	< 0.001
Nutrients					
Proteins (% of energy)	13.79 ± 0.08	14.62 ± 0.08	15.08 ± .08	15.77 ± 0.08	< 0.001
Fats (% of energy)	39.71 ± 0.23	38.19 ± 0.22	36.92 ± 0.22	35.25 ± 0.22	< 0.001
Carbohydrates (% of energy)	47.77 ± 0.28	48.60 ± 0.28	49.54 ± 0.28	50.60 ± 0.28	< 0.001
Dietary fiber (g/d)	20.70 ± 0.20	22.53 ± 0.20	23.40 ± 0.20	23.73 ± 0.20	< 0.001
Omega-3 fatty acids (g/d)	1.76 ± 0.02	1.77 ± 0.02	1.76 ± 0.02	1.67 ± 0.02	< 0.001
Vitamin B1 (mg/d)	1.55 ± 0.02	1.79 ± 0.02	1.94 ± 0.02	2.09 ± 0.02	< 0.001
Vitamin B6 (mg/d)	1.95 ± 0.01	2.00 ± 0.01	1.99 ± 0.01	1.98 ± 0.01	< 0.001
Iron (mg/d)	16.74 ± 0.12	17.64 ± 0.11	18.15 ± 0.11	17.89 ± 0.11	< 0.001
Vitamin C (mg/d)	88.78 ± 1.89	101.12 ± 1.86	104.48 ± 1.84	111.98 ± 1.85	< 0.001
Vitamin E (mg/d)	24.93 ± 0.19	22.60 ± 0.19	20.73 ± 0.19	17.66 ± 0.19	< 0.001
Food groups (g/d)					
Red meat	94.05 ± 1.42	85.37 ± 1.40	75.31 ± 1.39	60.22 ± 1.39	< 0.001
Whole grains	21.79 ± 2.75	36.42 ± 2.70	46.48 ± 2.68	64.68 ± 2.69	< 0.001
Refined grains	436.05 ± 6.02	398.75 ± 5.90	385.41 ± 5.86	353.12 ± 5.89	< 0.001
Fruit	280.85 ± 8.43	318.43 ± 8.27	328.74 ± 8.20	341.61 ± 8.25	< 0.001
Vegetables	210.56 ± 4.31	232.67 ± 4.22	248.40 ± 4.19	264.22 ± 4.21	< 0.001
Nuts, soy and legumes	63.98 ± 1.33	58.85 ± 1.30	57.69 ± 1.29	48.71 ± 1.30	< 0.001
Low fat dairy	152.94 ± 7.39	243.53 ± 7.25	334.64 ± 7.20	597.30 ± 7.24	< 0.001
High fat dairy	13.82 ± 0.64	15.23 ± 0.63	15.28 ± 0.62	14.45 ± 0.62	< 0.001

All values are means ± standard error (SE); energy intake is adjusted for age and sex; all other values are adjusted for age, sex and energy intake.

^a^Obtained from ANCOVA.

**Figure 1:**
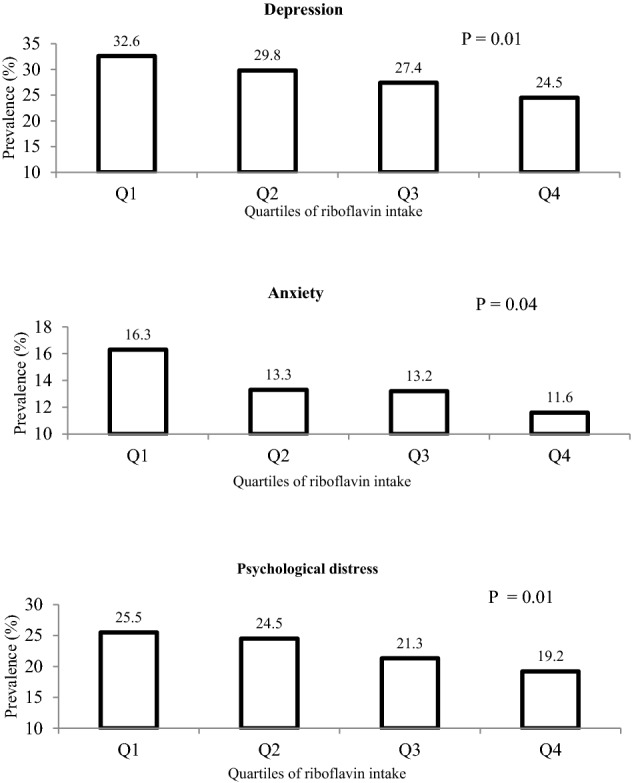
Prevalence of depression, anxiety and high psychological distress across energy-adjusted quartiles of dietary riboflavin intake.

As depicted in Fig. 1, participants in the top category of riboflavin intake compared to the bottom category had a significant lower prevalence of depression (24.5% vs. 32.6%; P = 0.01), anxiety (11.6% vs. 16.3%; P = 0.04), and distress (19.2% vs. 25.5%; P = 0.01).

Multivariable-adjusted odds ratios for depression, anxiety, and distress across quartiles of riboflavin intake are provided in Table 3. Compared to the lowest quartile, adults in the highest quartile of riboflavin intake had a 34% decreased odds of depression (OR = 0.66; 95%CI 0.54, 0.83). This association remained significant, even after adjusting for all potential confounders (OR = 0.66; 95%CI 0.49, 0.88). Greater intake of riboflavin was also associated with 33% lower odds of anxiety (OR = 0.67; 95%CI 0.48, 0.95). This relation strengthened after controlling for all cofounders (OR = 0.64; 95%CI 0.44, 0.94). Compared to the lowest quartile, individuals in the highest quartile of riboflavin intake had a decreased odds of distress either in crude (OR = 0.69, 95%CI 0.55, 0.87) or in fully-adjusted model (OR = 0.65, 95%CI 0.48, 0.89); such that, the top quartile of riboflavin intake in comparison to the bottom quartile was associated with 35% decreased odds of distress. A significant trend was additionally observed across quartiles of riboflavin intake and chance of all psychological disorders (P < 0.05 for all models).

**Table 3:** Multivariable-adjusted odds ratios and 95% confidence intervals for depression, anxiety and psychological distress across energy-adjusted quartiles of riboflavin intake in whole population (n = 3362).

	Quartiles of riboflavin intake				P_trend_
	Q1 (n = 840) (< 1.6 mg/d)	Q2 (n = 841) (1.6–1.82 mg/d)	Q3 (n = 841) (1.83–2.09 mg/d)	Q4 (n = 840) (> 2.09 mg/d)	
Depression					
Crude	1 (Ref.)	0.87 (0.71–1.08)	0.78 (0.63–0.96)	0.66 (0.54–0.83)	< 0.001
Model 1	1 (Ref.)	0.85 (0.68–1.07)	0.73 (0.57–0.91)	0.66 (0.52–0.83)	< 0.001
Model 2	1 (Ref.)	0.83 (0.65–1.05)	0.73 (0.58–0.93)	0.65 (0.51–0.83)	< 0.001
Model 3	1 (Ref.)	0.82 (0.64–1.05)	0.73 (0.56–0.94)	0.65 (0.49–0.86)	0.01
Model 4	1 (Ref.)	0.82 (0.63–1.05)	0.71 (0.55–0.92)	0.66 (0.49–0.88)	0.01
Anxiety					
Crude	1 (Ref.)	0.78 (0.59–1.03)	0.77 (0.59–1.02)	0.67 (0.51–0.89)	0.01
Model 1	1 (Ref.)	0.70 (0.52–0.95)	0.69 (0.51–0.93)	0.68 (0.51–0.92)	0.01
Model 2	1 (Ref.)	0.67 (0.49–0.92)	0.73 (0.53–0.99)	0.67 (0.49–0.93)	0.03
Model 3	1 (Ref.)	0.65 (0.47–0.91)	0.70 (0.50–0.97)	0.65 (0.45–0.94)	0.04
Model 4	1 (Ref.)	0.66 (0.47–0.92)	0.68 (0.48–0.95)	0.64 (0.44–0.94)	0.03
Psychological distress					
Crude	1 (Ref.)	0.94 (0.76–1.18)	0.79 (0.63–0.99)	0.69 (0.55–0.87)	0.01
Model 1	1 (Ref.)	0.95 (0.75–1.21)	0.78 (0.61–0.99)	0.75 (0.58–0.96)	0.01
Model 2	1 (Ref.)	0.92 (0.71–1.18)	0.78 (0.61–1.01)	0.73 (0.56–0.94)	0.01
Model 3	1 (Ref.)	0.87 (0.67–1.13)	0.73 (0.55–0.95)	0.66 (0.49–0.89)	0.01
Model 4	1 (Ref.)	0.87 (0.67–1.13)	0.71 (0.54–0.94)	0.65 (0.48–0.89)	0.01

Model 1: Adjusted for age, sex and energy intake; Model 2: Further adjustment for physical activity, smoking, marital status, education, household size, house possession, diabetes, use of anti-depressant medications and dietary supplements; Model 3: Additional controlling for dietary intakes of fat, n-3 fatty acids, iron, thiamin; Model 4: Further adjusted for BMI.

Multivariable-adjusted odds ratios for psychological disorders across different categories of riboflavin intake, stratified by sex, are presented in Table 4. After controlling all confounding variables, the highest level of riboflavin intake, compared to the lowest level, was respectively linked to 51% and 55% significant reduced odds of depression (OR = 0.49, 95%CI 0.29, 0.83) and anxiety (OR = 0.45, 95%CI 0.21, 0.95) in men. However, no relation was observed between intake of riboflavin and distress among men. Women in the highest quartile of riboflavin intake, compared to those in the lowest quartile, had a 33% significant decreased odds of distress (OR = 0.67, 95%CI 0.46, 0.98). However, no relation was observed between riboflavin intake and depression or anxiety disorders among female participants.

**Table 4:** Multivariable-adjusted odds ratios and 95% confidence intervals for depression, anxiety and psychological distress across energy-adjusted quartiles of riboflavin consumption, stratified by sex.

	Quartiles of riboflavin intake				P_trend_
	Q1 (< 1.6 mg/d)	Q2 (1.6–1.82 mg/d)	Q3 (1.83–2.09 mg/d)	Q4 (> 2.09 mg/d)	
Male participants (n = 1403)					
Depression					
Crude	1 (Ref.)	0.67 (0.47–0.95)	0.66 (0.46–0.95)	0.50 (0.34–0.72)	< 0.001
Model 1	1 (Ref.)	0.67 (0.45–1.00)	0.66 (0.44–1.00)	0.48 (0.32–0.73)	0.01
Model 2	1 (Ref.)	0.66 (0.43–1.00)	0.66 (0.43–1.01)	0.49 (0.32–0.76)	0.01
Model 3	1 (Ref.)	0.66 (0.43–1.01)	0.65 (0.42–1.02)	0.53 (0.32–0.87)	0.01
Model 4	1 (Ref.)	0.65 (0.42–1.01)	0.59 (0.37–0.94)	0.49 (0.29–0.83)	0.01
Anxiety					
Crude	1 (Ref.)	0.48(0.29–0.82)	0.57(0.34–0.95)	0.47(0.28–0.80)	0.01
Model 1	1 (Ref.)	0.44(0.24–0.81)	0.53(0.30–0.97)	0.46(0.25–0.83)	0.02
Model 2	1 (Ref.)	0.40(0.21–0.76)	0.50(0.27–0.93)	0.50(0.27–0.92)	0.03
Model 3	1 (Ref.)	0.39 (0.20–0.76)	0.51 (0.26–0.98)	0.48 (0.23–0.98)	0.05
Model 4	1 (Ref.)	0.42 (0.21–0.82)	0.47 (0.23–0.93)	0.45 (0.21–0.95)	0.03
Psychological distress					
Crude	1 (Ref.)	0.80 (0.55–1.16)	0.68 (0.46–1.01)	0.58 (0.39–0.87)	0.01
Model 1	1 (Ref.)	0.85 (0.56–1.31)	0.75 (0.48–1.16)	0.70 (0.45–1.08)	0.09
Model 2	1 (Ref.)	0.83 (0.53–1.30)	0.70 (0.44–1.12)	0.70 (0.43–1.10)	0.09
Model 3	1 (Ref.)	0.81 (0.51–1.27)	0.67 (0.41–1.09)	0.68 (0.40–1.15)	0.11
Model 4	1 (Ref.)	0.84 (0.52–1.34)	0.61 (0.37–1.02)	0.58 (0.33–1.01)	0.03
Female participants (n = 1959)					
Depression					
Crude	1 (Ref.)	1.02 (0.79–1.33)	0.82 (0.63–1.07)	0.78 (0.60–1.02)	0.03
Model 1	1 (Ref.)	0.95 (0.72–1.26)	0.76 (0.57–1.00)	0.76 (0.57–1.00)	0.02
Model 2	1 (Ref.)	0.93 (0.69–1.24)	0.76 (0.56–1.01)	0.72 (0.53–0.97)	0.01
Model 3	1 (Ref.)	0.921 (0.67–1.24)	0.74 (0.54–1.02)	0.70 (0.50–1.00)	0.02
Model 4	1 (Ref.)	0.90 (0.66–1.23)	0.75 (0.54–1.03)	0.74 (0.52–1.06)	0.06
Anxiety					
Crude	1 (Ref.)	0.96 (0.69–1.33)	0.86 (0.62–1.20)	0.79 (0.56–1.10)	0.14
Model 1	1 (Ref.)	0.83(0.59–1.17)	0.76 (0.54–1.07)	0.79(0.55–1.11)	0.14
Model 2	1 (Ref.)	0.80 (0.55–1.16)	0.80 (0.55–1.16)	0.76 (0.52–1.10)	0.17
Model 3	1 (Ref.)	0.76 (0.51–1.11)	0.77 (0.52–1.13)	0.74 (0.47–1.14)	0.20
Model 4	1 (Ref.)	0.75 (0.50–1.11)	0.75 (0.50–1.12)	0.74 (0.47–1.15)	0.21
Psychological distress					
Crude	1 (Ref.)	1.04 (0.79–1.37)	0.82 (0.62–1.09)	0.75 (0.56–1.00)	0.02
Model 1	1 (Ref.)	1.00 (0.75–1.34)	0.78 (0.58–1.05)	0.77 (0.57–1.04)	0.03
Model 2	1 (Ref.)	0.96 (0.70–1.30)	0.80 (0.59–1.08)	0.74 (0.54–1.01)	0.03
Model 3	1 (Ref.)	0.89 (0.65–1.22)	0.71 (0.52–1.00)	0.63 (0.44–0.91)	0.01
Model 4	1 (Ref.)	0.87 (0.63–1.21)	0.71 (0.51–1.00)	0.67 (0.46–0.98)	0.02

All values are odds ratios and 95% confidence intervals. Model 1: Adjusted for age and energy intake; Model 2: Further adjustment for physical activity, smoking, marital status, education, household size, house possession, diabetes, use of anti-depressant medications and dietary supplements; Model 3: Additional controlling for dietary intakes of fat, n-3 fatty acids, iron, thiamin; Model 4: Further adjusted for BMI.

Multivariable-adjusted odds ratios for psychological disorders across quartiles of riboflavin intake, stratified by BMI categories, are reported in Table 5. In normal-weight participant (BMI < 25 kg/m^2^), the top quartile of riboflavin intake compared to the bottom quartile was associated with a significant 38% decrease in depression odds (OR = 0.62, 95%CI 0.41, 0.94), in fully-adjusted model. Also, participants in the top quartile of riboflavin intake were marginally less likely to have distress, in comparison to those in the bottom quartile (OR = 0.66, 95%CI 0.41, 1.02). Among overweight/obese individuals (BMI ≥ 25 kg/m^2^), no association was observed between riboflavin consumption and anxiety in fully-adjusted model. The highest level of riboflavin intake, as compared to the lowest level, was associated with a marginally significant decrease in odds of depression, in fully adjusted model (OR = 0.66; 95% CI 0.44, 1.00). Higher riboflavin intake was also associated with a 42% decreased odds of distress (OR = 0.58; 95% CI 0.38, 0.90), after adjustment for confounders.

**Table 5:** Multivariable-adjusted odds ratios and 95% confidence intervals for depression, anxiety and high psychological distress across energy-adjusted quartiles of riboflavin intake, stratified by BMI.

	Quartiles of riboflavin intake				P_trend_
	Q1 (< 1.6 mg/d)	Q2 (1.6–1.82 mg/d)	Q3 (1.83–2.09 mg/d)	Q4 (> 2.09 mg/d)	
Normal weight participants (BMI < 25 kg/m^2^) (n = 1856)					
Depression					
Crude	1 (Ref.)	0.99 (0.74–1.31)	0.74 (0.55–1.00)	0.63 (0.46–0.86)	0.01
Model 1	1 (Ref.)	1.00 (0.74–1.36)	0.72 (0.52–0.98)	0.67 (0.48–0.94)	0.01
Model 2	1 (Ref.)	0.96 (0.70–1.33)	0.74 (0.53–1.03)	0.64 (0.45–0.92)	0.01
Model 3	1 (Ref.)	0.94 (0.67–1.32)	0.71 (0.50–1.01)	0.62 (0.41–0.94)	0.01
Anxiety					
Crude	1 (Ref.)	0.97 (0.66–1.43)	0.97 (0.66–1.43)	0.63 (0.40–0.98)	0.07
Model 1	1 (Ref.)	0.98 (0.65–1.47)	0.88 (0.58–1.33)	0.66 (0.41–1.04)	0.08
Model 2	1 (Ref.)	0.94 (0.60–1.46)	1.01 (0.66–1.57)	0.64 (0.39–1.06)	0.17
Model 3	1 (Ref.)	0.91 (0.58–1.44)	0.98 (0.61–1.55)	0.63 (0.35–1.11)	0.21
Psychological distress					
Crude	1 (Ref.)	1.13 (0.84–1.52)	0.77 (0.56–1.05)	0.64 (0.46–0.90)	0.01
Model 1	1 (Ref.)	1.19 (0.86–1.64)	0.76 (0.54–1.07)	0.71 (0.50–1.01)	0.01
Model 2	1 (Ref.)	1.14 (0.81–1.61)	0.79 (0.56–1.13)	0.73 (0.50–1.06)	0.03
Model 3	1 (Ref.)	1.09 (0.76–1.55)	0.73 (0.50–1.07)	0.66 (0.43–1.02)	0.02
Overweight/obese participants (BMI ≥ 25 kg/m^2^) (n = 1506)					
Depression					
Crude	1 (Ref.)	0.79 (0.57–1.09)	0.84 (0.61–1.16)	0.73 (0.54–1.00)	0.09
Model 1	1 (Ref.)	0.73 (0.51–1.04)	0.73 (0.51–1.04)	0.66 (0.47–0.92)	0.03
Model 2	1 (Ref.)	0.72 (0.50–1.04)	0.72 (0.50–1.04)	0.65 (0.45–0.92)	0.03
Model 3	1 (Ref.)	0.73 (0.50–1.06)	0.73 (0.50–1.08)	0.66 (0.44–1.00)	0.07
Anxiety					
Crude	1 (Ref.)	0.66 (0.43–0.99)	0.64 (0.42–0.97)	0.68 (0.46–1.00)	0.06
Model 1	1 (Ref.)	0.51 (0.32–0.82)	0.54 (0.34–0.85)	0.66 (0.43–1.00)	0.09
Model 2	1 (Ref.)	0.50 (0.31–0.82)	0.52 (0.32–0.85)	0.66 (0.42–1.03)	0.11
Model 3	1 (Ref.)	0.49 (0.30–0.81)	0.50 (0.30–0.83)	0.61 (0.36–1.01)	0.07
Psychological distress					
Crude	1 (Ref.)	0.77 (0.54–1.09)	0.81 (0.57–1.15)	0.75 (0.54–1.05)	0.15
Model 1	1 (Ref.)	0.73 (0.50–1.06)	0.78 (0.54–1.14)	0.77 (0.54–1.10)	0.25
Model 2	1 (Ref.)	0.67 (0.45–1.00)	0.74 (0.50–1.09)	0.68 (0.46–0.99)	0.09
Model 3	1 (Ref.)	0.64 (0.42–0.96)	0.68 (0.45–1.02)	0.58 (0.38–0.90)	0.03

All values are odds ratios and 95% confidence intervals. Model 1: Adjusted for age, sex and energy intake; Model 2: Further adjustment for physical activity, smoking, marital status, education, household size, house possession, diabetes, use of anti-depressant medications and dietary supplements; Model 3: Additional controlling for dietary intakes of fat, n-3 fatty acids, iron, thiamin.

## Discussion

This population-based study revealed that dietary riboflavin intake was inversely linked to psychological disorders in Iranian adults. This association was independent from several potential confounders. Moreover, stratified analysis revealed that greater riboflavin intake from diet was linked to lower chance of depression and anxiety in men. In addition, an inverse association was seen between dietary intake of riboflavin and psychological distress in women. In addition, the associations between riboflavin intake and depression and psychological distress were independent from BMI categories in adults. To our knowledge, this is one of the first studies which investigated the association between dietary intake of riboflavin and psychological disorders in Iranian adults.

The prevalence of depression, anxiety, and other psychological disorders has dramatically risen across the world^1,3^. Along with other chronic conditions such as obesity^45^, cardiovascular disease^46^, diabetes^47^, and cancers^48^, these mental disorders have become global health issues and imposed substantial economic burden on healthcare systems^49^. As a result, prevention strategies for these disorders are crucial^50^. The findings of current study suggested that high intake of riboflavin from foods might be helpful for general adult population to prevent these conditions. People should be advised to consume more dietary sources of riboflavin such as dairy products, leafy vegetables, legumes, liver, kidneys, yeast, and mushrooms^51^.

Some prior studies have reported an inverse association between vitamin B2^52,53^ and other B-vitamins with psychological disorders in various nations^28,29^. The current study showed that higher intake of riboflavin was protectively related to lower chance of depression. Similarly, a cross-sectional study in adult population revealed a reduced odds of depression in relation to the highest intake of riboflavin^53^, although stratified analysis based on sex was not conducted in the mentioned study. Another cross-sectional investigation on psychiatric in-patients showed a significant inverse relationship between riboflavin intake and endogenous depression^52^. In addition, a cross-sectional study on 6517 adolescents demonstrated that riboflavin intake was inversely associated with depressive symptoms in girls, but not in boys^30^. In line with our findings, a meta-analysis on six epidemiologic investigations revealed a significant inverse linkage between riboflavin intake and depression; however, five of these investigations were conducted in East Asian countries^54^. A cross-sectional study on 314 HIV-infected adults revealed that about 26% of participants suffered from depression^55^. More than 67% of individuals consumed B-vitamins less than estimated average requirements (EAR) and low consumption of riboflavin was connected to depression risk in women, but not in men. Although the exact mechanism remains unclear, it was proposed that female gonadal hormones might change the serotoninergic activity in the brain, alter regulation of monoamines levels such as serotonin, and result in higher levels of depressive symptoms^56^. However, stratified analysis in the current study revealed a significant inverse relation between dietary riboflavin intake and depression in men, but not in women.

Subgroup analysis by sex has additionally documented the inverse association only in women. A prospective birth-cohort study conducted on 636 British women has documented a non-significant inverse relationship between riboflavin intake and psychological distress. In the mentioned cohort GHQ-28, multiple 24-h recalls and a 5-day food record were applied to measure psychological distress and dietary vitamin B2 assessment^57^. More prospective investigations in this regard are needed.

A possible concern in interpreting findings of epidemiologic studies is reverse causation, particularly when the study design is cross-sectional. The reverse causation refers to the fact that symptoms of depression might lead to changes in dietary intakes of individuals^58^. Such that, a longitudinal investigation demonstrated the existence of bidirectional relationships between food intakes and depression^59^, especially in case of meat, dairy products, and vegetable intake^59^.

The analysis of the current study revealed significant differences in some nutrients and vitamins between categories of riboflavin intake. In other words, those who had more riboflavin intake also had more other nutrients and vitamins intake or had a healthier dietary pattern. Therefore, this healthier dietary intake might result in reduction of psychological disorders odds.

Several probable mechanisms might elucidate the observed associations between vitamin B2 and mental health. FMN and FAD are two crucial rate-limiting flavoprotein coenzymes which derive from riboflavin^60^. Flavoproteins are co-factors in the metabolism of essential fatty acids in brain lipids^51^. The action of riboflavin coenzymes in the re-methylation and trans-sulphuration of homocysteine may also explain the benefits of riboflavin on mental health^61,62^, as an inverse relation among homocysteine and circulating concentrations of riboflavin was previously reported in National Health and Nutrition Examination Survey (NHANES) and Framingham Offspring cohort^63,64^. Thus, high level of homocysteine may mediate the connection between riboflavin deficiency and high risk of depression.

The current study has several strengths. A considerable population of adults was investigated by the use of validated questionnaires for assessment of dietary intakes, physical activity, and psychological disorders. Several potential confounders were also considered in the current analyses. However, some limitations should be acknowledged when interpreting the findings. This study cannot infer a causal relationship between riboflavin intake and psychological disorders due to the cross-sectional design of the study. Further studies with prospective design are needed to establish causality. The study was conducted on non-academic personnel of a medical university with different socioeconomic levels; although the sample was somehow representative of general adult population, extrapolating our findings to other populations should be done with caution. Finally, although a validated self-administered FFQ was applied, misclassification of subjects was inevitable.

In conclusion, the current study found that dietary riboflavin intake was inversely associated with chance of psychological disorders in Iranian adults. High intake of riboflavin decreased the chance of depression and anxiety in men and high psychological distress in women. More prospective studies are needed to confirm these findings.

## Supplementary Information

Supplementary Figure 1.

## Data Availability

The data that support the findings of this study are available from the corresponding author [PS], upon reasonable request.

## Abbreviations

GPPAQ: General Practice Physical Activity
GHQ: General Health Questionnaire
IPAQ: International Physical Activity Questionnaire
ANOVA: Analysis of variance
FFQ: Food frequency questionnaire
HADS: Hospital Anxiety and Depression Scale
OR: Odds ratios
SD: Standard deviation
95% CI: 95% Confidence interval
ANCOVA: Analysis of covariance
SE: Standard error
EAR: Estimated average requirement
