# Supplementary material for: Dietary riboflavin intake in relation to psychological disorders in Iranian adults: an observational study
Source: Sci Rep. 2023 Mar 29;13:5152. doi: 10.1038/s41598-023-32309-w (PMC10060244; doi:10.1038/s41598-023-32309-w)
Supplement: Supplementary file 1 — Supplementary Figure 1. [file 41598_2023_32309_MOESM1_ESM.pptx]

## Slide 1
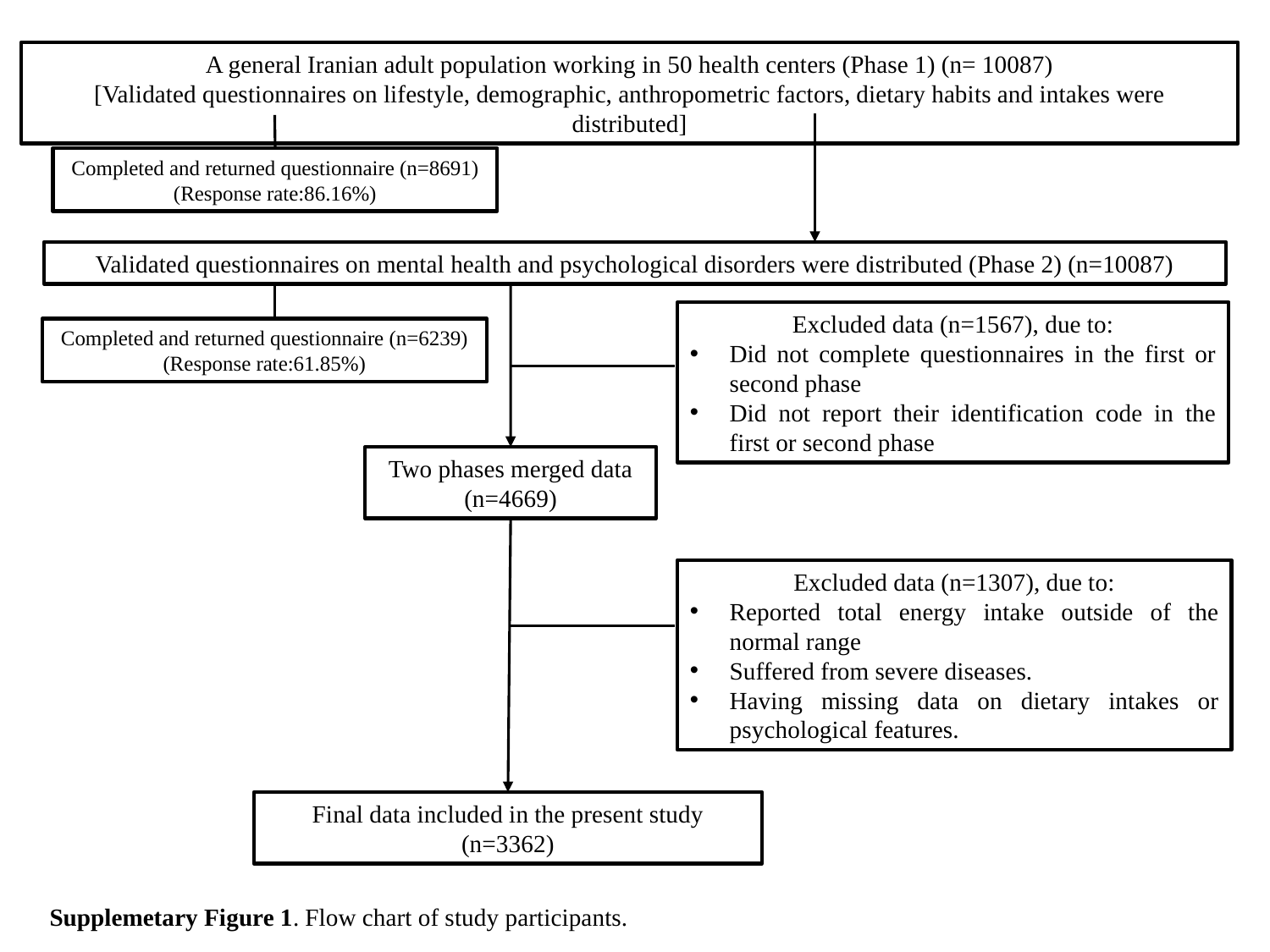

A general Iranian adult population working in 50 health centers (Phase 1) (n= 10087)
[Validated questionnaires on lifestyle, demographic, anthropometric factors, dietary habits and intakes were distributed]
Completed and returned questionnaire (n=8691)
(Response rate:86.16%)
Validated questionnaires on mental health and psychological disorders were distributed (Phase 2) (n=10087)
Excluded data (n=1567), due to:
Did not complete questionnaires in the first or second phase
Did not report their identification code in the first or second phase
Completed and returned questionnaire (n=6239)
(Response rate:61.85%)
Two phases merged data
(n=4669)
Excluded data (n=1307), due to:
Reported total energy intake outside of the normal range
Suffered from severe diseases.
Having missing data on dietary intakes or psychological features.
Final data included in the present study (n=3362)
Supplemetary Figure 1. Flow chart of study participants.
